# Behavioral and Molecular Consequences of Chronic Sleep Restriction During Development in Fragile X Mice

**DOI:** 10.3389/fnins.2022.834890

**Published:** 2022-06-27

**Authors:** R. Michelle Saré, Alex Song, Merlin Levine, Abigail Lemons, Inna Loutaev, Carrie Sheeler, Christine Hildreth, Angel Mfon, Carolyn Beebe Smith

**Affiliations:** Section on Neuroadaptation and Protein Metabolism, Department of Health and Human Services, National Institute of Mental Health, National Institutes of Health, Bethesda, MD, United States

**Keywords:** chronic sleep restriction, gentle handling, fragile X, social behavior, autism, mTOR, myelin

## Abstract

Sleep is critical for brain development and synaptic plasticity. In male wild-type mice, chronic sleep restriction during development results in long-lasting impairments in behavior including hypoactivity, decreased sociability, and increased repetitive behavior. Disordered sleep is characteristic of many neurodevelopmental disorders. Moreover, the severity of behavioral symptoms is correlated with the degree of disordered sleep. We hypothesized that chronic developmental sleep restriction in a mouse model of fragile X syndrome (FXS) would exacerbate behavioral phenotypes. To test our hypothesis, we sleep-restricted *Fmr1* knockout (KO) mice for 3 h per day from P5 to P52 and subjected mice to behavioral tests beginning on P42. Contrary to our expectations, sleep restriction improved the hyperactivity and lack of preference for social novelty phenotypes in *Fmr1* KO mice but had no measurable effect on repetitive activity. Sleep restriction also resulted in changes in regional distribution of myelin basic protein, suggesting effects on myelination. These findings have implications for the role of disrupted sleep in the severity of symptoms in FXS.

## Introduction

Sleep is important for brain development and synaptic plasticity (Peirano and Algarin, 2007). Sleep problems accompany numerous neurodevelopmental disorders including autism spectrum disorders (ASDs) and fragile X syndrome (FXS) (Picchioni et al., 2014). Moreover, sleep problems correlate with the severity of behavioral problems (Kronk et al., 2010). Previously, we found that chronic sleep restriction during development altered behavioral trajectories in wild-type (WT) mice in a sex-specific manner (Sare et al., 2015). In particular, chronic sleep restriction resulted in long-lasting hypoactivity (both sexes) and a reduced preference for social novelty in males (Sare et al., 2019). These results suggest, at least in WT mice, that sleep restriction during development alters behavior in adulthood and in males, results in impaired social behavior.

The mouse model for FXS, *Fmr1* knockout (KO), exhibits some behaviors typical of ASDs including abnormal social behavior and reduced sleep (Liu and Smith, 2009; Kazdoba et al., 2014; Sare et al., 2017a,b; Boone et al., 2018). *Fmr1* KO mice also display hyperactivity in the open field, decreased anxiety-like behaviors, repetitive behaviors, and learning and memory impairments (Liu et al., 2011; Kazdoba et al., 2014; Sare et al., 2017b). We hypothesized that sleep problems might be contributing to some of the behavioral problems in *Fmr1* KO mice, including social behavior abnormalities. In keeping with this idea, exacerbating sleep problems by performing chronic sleep restriction during development would be expected to yield more severe behavioral impairments.

We chronically sleep-restricted male *Fmr1* KO mice and determined the effect on open field activity, anxiety-like behavior, repetitive behavior, sleep duration, and social behavior. Contrary to our expectations, sleep restriction improved the hyperactivity and social behavior phenotypes in *Fmr1* KO mice, but other behavioral phenotypes were not affected. Sleep restriction also resulted in changes in regional distribution of myelin basic protein (MBP), suggesting effects on myelination. These findings have implications for the role of disrupted sleep on the severity of symptoms in FXS.

## Materials and Methods

### Animals

*Fmr1* KO animals (on a C57BL/6J background) were generated in-house by mating WT males with heterozygous females. We periodically backcrossed C57BL/6J mice back into the colony to maintain the background. At 10 days of age, animals were ear punched for identification, and the ear punch was used for the determination of genotypes as previously described (Qin et al., 2005). Animals were housed in a central facility with a standard 12:12 light/dark cycle (lights on at 6 AM) with *ad libitum* access to food and water. All procedures were approved by the National Institute of Mental Health Animal Care and Use Committee and were performed according to the National Institutes of Health Guidelines on the Care and Use of Animals.

### Study Design

We studied three groups of male *Fmr1* KO mice, namely, (1) controls, mice permitted to sleep *ad libitum* in the animal housing room; (2) sleep restriction, mice subjected to daily (3 h/day) sleep restriction by gentle handling commencing on postnatal day 5 (P5); and (3) stress, mice subjected to daily gentle prodding every 15 min for 3 h/day commencing on P5. Concurrently, we also studied WT mice, but we have published these results separately (Sare et al., 2019). In this article, we compared results in WT and *Fmr1* KO mice including some statistical analyses. Once a dam gave birth, the entire litter was assigned to one of the three groups. For the control and stress groups, we had 12 litters represented, and for the sleep restriction groups, we had 14 litters. Each group, therefore, included one-two mice from each litter. Dams in either the sleep-restricted or the stress group provided only one litter for the study. When the pups were 5 days of age, the sire of the cage was removed. Sleep restriction and stress procedures occurred in a separate room for 3 h/day (11:00 AM–2:00 PM) until P52. Sleep restriction was performed by gentle handling as previously described (Sare et al., 2015; Lemons et al., 2018). Mice in the stress group were gently prodded with a small paint brush once every 15 min from 11:00 AM to 2:00 PM daily until P52. This frequency of prodding allowed mice in the stress group minimal interruption of sleep.

### Behavior Testing

Behavior testing was initiated at P42, and tests were spaced 2 days apart. Behavior testing was conducted between 11:00 AM and 2:00 PM and occurred instead of sleep restriction or stress procedures. Testing was performed in the following order: open field, marble burying, social behavior, and social transmission of food preference (not presented). All animals underwent all tests, though for reasons listed below, not all data could be included for all tests. Following sleep restriction or stress, mice were allowed 4 weeks of recovery with *ad libitum* sleep. During this time, we measured sleep duration. After the recovery period, we conducted the same battery of behavior tests as previously without additional sleep restriction or stress. The timeline of sleep restriction or stress and behavior testing is shown in Table 1. In all behavior tests, the experimenter left the room during the test.

**TABLE 1:** Experiment schedule.

Postnatal age (days)	Procedure
5–52	Sleep-restriction
42	Open field
45	Marble burying
48	Social behavior
50–52	Social transmission of food preference
52–84	Recovery sleep
74–77	Sleep testing
84	Open field
87	Marble burying
90	Social behavior
92–94	Social transmission of food preference
94	Harvest for Western blotting

### Open Field

Open-field testing was conducted as previously described by means of the Colbourn TruScan system (Colbourn Instruments, Whitehall, PA, United States) (Sare et al., 2015). Animals were placed in the novel environment for 30 min, and activity was assessed in six epochs, each lasting 5 min. Activity was detected by beam breaks and determined by analysis of the total horizontal distance traveled. Anxiety-like behavior was assessed by comparing the ratio of distance traveled in the center (more than 6.25 cm from the wall) to the total distance traveled during each 5 min epoch. Sometimes, during the testing, equipment failed and did not record the data. Since the animal had already completed the behavior test, it could not be run again as the environment was no longer novel.

### Marble Burying

Marble burying was performed as previously described (Sare et al., 2015). A grid of 20 glass marbles was overlain on top of hardwood bedding (4.5 cm in depth) in a testing cage, and the mouse was placed in the cage. After 30 min, the mouse was removed, and the number of marbles buried (>50%) was assessed.

### Social Behavior

Social behavior was analyzed by means of the standard three-chambered apparatus as previously described (Sare et al., 2015). Time in chamber was assessed by beam breaks. Time sniffing was assessed by the analysis using TopScan (Clever Systems, Reston, VA, United States). Parameters were set to define sniffing if an animal was within 20 mm of the enclosure with his nose directed toward it. Each test period was broken into three phases (5 min each). (1) Habituation to the empty chamber. If an animal spent more than 3 min in any chamber, or avoided entry into a chamber during this period, it was excluded from the study. (2) Sociability: on one side of the chamber, a novel age/sex-matched stranger mouse (WT mouse on a C57BL/6J background) was placed in a sociability cup (Noldus, Leesburg, VA, United States). On the opposite side, an empty sociability cup was placed. (3) Preference for social novelty: a novel age/sex-matched stranger mouse was placed into the previously empty sociability cup from phase 2. This was now defined as the novel mouse. During some of the tests, equipment failed or the operator made errors; these data were excluded.

### Social Transmission of Food Preference (Data Not Shown)

We randomly chose one mouse from each cage to be the demonstrator mouse. The demonstrator mouse was separated from his littermates, singly housed, and food-deprived overnight. He was then given food with either 2% cocoa or 1% cinnamon for 1 h. If he consumed at least 0.2 g, he was then reintroduced to his littermates for them to interact for 30 min. The cage mates were food-deprived for 24 h, then each mouse was singly housed for 1 h and given a choice between the cocoa and cinnamon-flavored foods. The amount of food consumed was assessed to determine preference. For the second round of testing, new flavors (1% cloves and 1% onion) were used, but the procedure remained the same. These data are not presented. In our hands, even control WT mice did not show the expected preference for the demonstrated food (Sare et al., 2019), indicating an inherent problem with the way the test was conducted. We include the description because all mice were subjected to this test, and administration of the test may have influenced subsequent tests.

### Sleep Testing

To determine sleep duration, we used home-cage monitoring *via* the activity monitoring system (CLAMS) (Columbus Instruments, Columbus, OH, United States) as previously described (Sare et al., 2017a,^2018^). Briefly, mice were singly housed and placed in the monitoring system for 72 h. We excluded the first 24 h of recording to allow for habituation to single housing and the new cage (Sare et al., 2017a). We averaged the data from the remaining 48 h. Activity was measured in 10 s epochs, and 40 s of consecutive inactivity was regarded as a bout of sleep as previously validated (Pack et al., 2007). Occasionally, the equipment failed, and we were unable to collect the data.

### Western Blotting

After the testing was completed, unanesthetized mice were decapitated; brains were rapidly dissected on ice into cerebellum, frontal cortex, striatum, thalamus, hippocampus, and parietal cortex; and tissues were placed into preweighed Precellys lysis tubes (Bertin Corporation, Rockville, MD, United States) and stored at −80°C. All mice were euthanized between 2:00 and 3:00 PM.

Tissues were later thawed at 4°C and homogenized in Tissue Protein Extraction Reagent solution (T-PER) (Thermo Scientific, Waltham, MA, United States) with 1% 0.5 M EDTA (Thermo Scientific, Waltham, MA, United States) and 1% Halt Protease and Phosphatase Inhibitor Cocktail (Thermo Scientific, Waltham, MA, United States) using the Precellys Homogenizer (Bertin Corporation, Rockville, MD, United States). Protein concentrations were determined by means of a Pierce BCA Protein Assay Kit (Thermo Scientific, Waltham, MA, United States). Extracted tissue protein (10 μg) was electrophoresed on a Bio-Rad mini-protein stain-free gel (Bio-Rad, Hercules, CA, United States). Protein was then transferred onto nitrocellulose membranes and exposed to primary antibody overnight at 4°C. The membrane was then incubated with secondary antibody (goat anti-rabbit horseradish peroxidase-linked) at 1:10,000 (Bio-Rad) for 1 h at room temperature. The membrane was then exposed to Clarity substrate (Bio-Rad, Hercules, CA, United States) and imaged *via* a chemiluminescent signal on a ChemiDoc MP Imager (Bio-Rad, Hercules, CA, United States). Total protein in the lane as determined by Bio-Rad stain-free technology was used for normalization.

Primary antibodies were diluted 1:1000 and were as follows: eukaryotic translation initiation factor 4E-binding protein 1 (p4EBP1) (Cell Signaling 9455), protein kinase B (pAKT) (Cell Signaling 4060), AMP-activated protein kinase (p-AMPK) (Cell Signaling 2535), circadian locomotor output cycles kaput (CLOCK) (Bethyl A302-618A), cAMP response element-binding protein (pCREB) (Cell Signaling 9198), extracellular regulated kinase (pERK) (Cell Signaling 3370), glycogen synthase kinase 3a/b (p-GSK3a/b) (Cell Signaling 9931), ionized calcium-binding adapter molecule 1 (Iba) (Abcam AB48004), C-Jun N-terminal kinase (pJNK) (Cell Signaling 9251), microtubule-associated protein 1A/1B light chain 3 (LC3) (Abgent AP1802a), MBP (Proteintech 10458-1-AP), pmTOR Ser2448 (mammalian target of rapamycin) (Cell Signaling 5536), pmTOR Thr2446 (Millipore 09345), p-p70 S6K (ribosomal protein S6 kinase) (Cell Signaling 9234), p-p90 RS6K (Cell Signaling 9335), p-S6 (ribosomal protein S6) 235/236 (Cell Signaling 2211), and p-S6 240/244 (Cell Signaling 2215).

### Corticosterone Analysis

Separate groups of animals were sleep-restricted or subjected to chronic stress according to the procedures described above from either P5 to P9 or from P5 to P42. Within 1 h of the sleep restriction period, unanesthetized animals were decapitated, and serum was collected and stored at −80°C. The serum was thawed and diluted 1:200 and processed with the ^125^I Corticosterone Radioimmunoassay Kit (MP Biomedicals, Solon, OH, United States) according to the manufacturer’s instructions.

### Statistical Analysis

The data are reported as means ± standard errors of the means (SEMs). Corticosterone concentrations were analyzed by means of a two-way ANOVA with condition (control, stress, or sleep restriction) and age (P9, P42) as between-subject variables. Open field and sleep duration were analyzed by means of a mixed model repeated measures ANOVA with condition (control, stress, or sleep restriction) as a between-subject variable and age (pre- or post-recovery) and either epoch (open field) or phase (sleep) as within-subject variables (SPSS, IBM Armonk, NY, United States). Social behavior was analyzed by means of paired *t*-tests. Sniffing times [stranger mouse vs. object (sociability) and novel mouse vs. familiar mouse (social novelty)] were compared both pre- and post-recovery. Marble burying was analyzed by means of a mixed model ANOVA with condition (control, stress, or sleep restriction) as a between-subject variable and age as a within-subject variable. Western blot results were analyzed by means of a one-way ANOVA with condition as the only variable. When appropriate, Bonferroni-corrected *post hoc* comparisons were performed. In the regional analysis for p-AMPK, MBP, and p-S6 in several cases (two for each protein), gel lanes were smudged, and we could not analyze a sample. In these cases, for the ANOVA, we substituted the mean value of the group for that point. The details are given in the figure legend. In this article, we also included some statistical analyses in which we included genotype WT, *Fmr1* KO as a variable (Supplementary Table 1). These analyses compared the results reported in this study with our previously reported results (Sare et al., 2019). Measurements in WT and *Fmr1* KO mice were contemporaneous.

## Results

### Growth Rate

**FIGURE 1:**
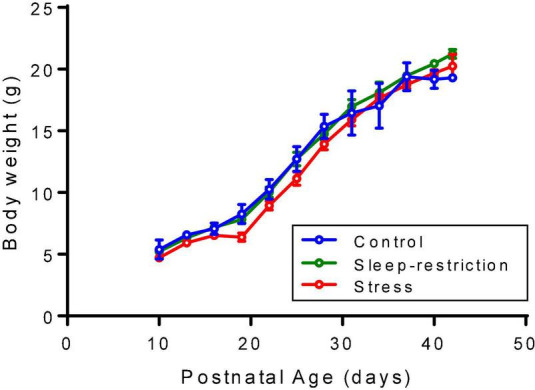
Mice were weighed every 3 days beginning at P10. Each point is the mean ± SEM for 2–5 controls, 2–5 sleep-restricted mice, and 2–11 stress mice. The exact number of mice at each age is designated for the three groups (i.e., control, sleep-restricted, and stress) as follows: P10 (3, 3, 5); P13 (3, 5, 11); P16 (5, 5, 11); P19 (4, 3, 9); P22 (5, 5, 11); P25 (5, 5, 7); P28 (5, 5, 11); P31 (5, 4, 11); P34 (5, 2, 11); P37 (5, 5, 11); P40 (3, 5, 11); and P42 (2, 4, 2). Data were analyzed by means of ANOVA with the condition as a between-subject factor and age as a within-subject factor. Condition/age mean values were substituted for missing values. Neither the condition × age interaction [*F*_(22_,_198)_ = 1.563, *p* = 0.0581] nor the main effect of condition [*F*_(2_,_198)_ = 0.1942, *p* = 0.1942] was statistically significant, but the main effect of age [*F*_(11_,_198)_ = 590.3, *p* < 0.0001] was, indicating that mice gained weight regardless of condition.

Every 3 days, beginning at P10, mice were weighed to assess the possible effects of procedures on growth. Neither sleep restriction nor stress impaired growth of the animals (Figure 1).

### Corticosterone Levels

**FIGURE 2:**
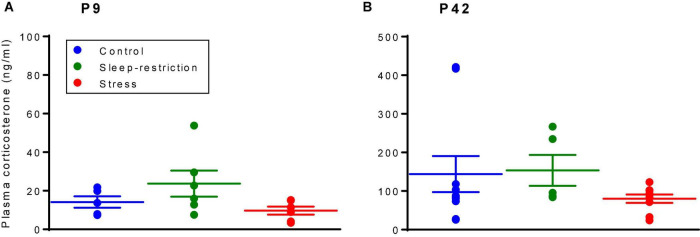
Corticosterone levels at P9 **(A)** and P42 **(B)** were determined by means of a radioimmunoassay. The main effect of age was statistically significant (*p* < 0.001); mice at P42, regardless of the condition, had higher serum corticosterone levels than mice at P9. The condition × age interaction and main effect of condition were not statistically significant. Each point represents the serum corticosterone concentration in a single animal. Lines represent means ± SEMs for 5 control, 6 stress, and 6 sleep-restricted mice at P9 and 10 control, 9 stress, and 5 sleep-restricted mice at P42.

As a measure of the stress level, we determined serum corticosterone concentrations after the three treatments described above (i.e., control, stress, and sleep restriction) at two different time points, namely, P9 and P42 (Figure 2). The main effect of age was statistically significant (*p* < 0.001) (Table 2), but the main effect of condition and the age × condition interaction were not. These results indicate that serum corticosterone concentrations were higher in the adult animals regardless of condition and that we did not detect an effect of either stress or sleep restriction.

**TABLE 2:** Repeated measures ANOVA results: behavioral data.

Test	Interaction	Main effect	*F*_(df,error)_	*p*-Value	Partial η^2^
Corticosterone	Age × condition		*F*_(2_,_35)_ = 0.581	0.565	0.032
		Age	*F*_(1_,_35)_ = 16.90	<0.001*	0.326^‡^
		Condition	*F*_(2_,_35)_ = 1.016	0.373	0.055
**Sleep**					
Total sleep time	Condition × phase		*F*_(2_,_63)_ = 1.186	0.312	0.036
		Condition	*F*_(2_,_63)_ = 2.379	0.101	0.070^†^
		Phase	*F*_(1_,_63)_ = 644.31	<0.001*	0.911^‡^
**Open field**					
Total distance moved	Age × condition × epoch		*F*_(8_,_223)_ = 1.167	0.320	0.040
	Condition × epoch		*F*_(7_,_207)_ = 3.874	<0.001*	0.122^†^
	Age × epoch		*F*_(4_,_223)_ = 2.244	0.066^∼^	0.039
	Age × condition		*F*_(2_,_56)_ = 1.565	0.218	0.053
		Age	*F*_(1_,_56)_ = 9.472	0.003*	0.145^‡^
		Condition	*F*_(2_,_56)_ = 6.751	0.002*	0.194^‡^
		Epoch	*F*_(4_,_207)_ = 104.87	<0.001*	0.652^‡^
Center/total ratio	Age × condition × epoch		*F*_(8_,_218)_ = 1.181	0.313	0.040
	Condition × epoch		*F*_(8_,_210)_ = 1.950	0.059^∼^	0.065^†^
	Age × epoch		*F*_(4_,_218)_ = 0.762	0.548	0.013
	Age × condition		*F*_(2_,_56)_ = 2.300	0.110	0.076^†^
		Age	*F*_(1_,_56)_ = 27.78	<0.001*	0.332^‡^
		Condition	*F*_(2_,_56)_ = 0.565	0.572	0.020
		Epoch	*F*_(4_,_210)_ = 12.56	<0.001*	0.183^‡^
**Marble burying**					
Buried	Age × condition		*F*_(2_,_71)_ = 1.445	0.242	0.039
		Age	*F*_(1_,_71)_ = 75.88	<0.001*	0.517^‡^
		Condition	*F*_(2_,_71)_ = 0.404	0.669	0.011

*p ≤ 0.05.

*∼, 0.10 ≥ p ≥ 0.05.*

*^†^Medium effect size based on partial η^2^.*

*^‡^Large effect size based on partial η^2^.*

For comparison of the effects of stress and sleep restriction on corticosterone levels in WT and *Fmr1* KO mice, we analyzed the two age groups separately (Supplementary Table 1) and found no main effect of genotype at P9 [*F*_(1_,_29)_ = 0.669, *p* = 0.410], but at P42, the main effect of genotype [*F*_(1_,_51)_ = 7.081, *p* = 0.010] was statistically significant. Regardless of the condition, *Fmr1* KO mice had higher levels of corticosterone at P42 than WT (Sare et al., 2019).

### Sleep Duration

**FIGURE 3:**
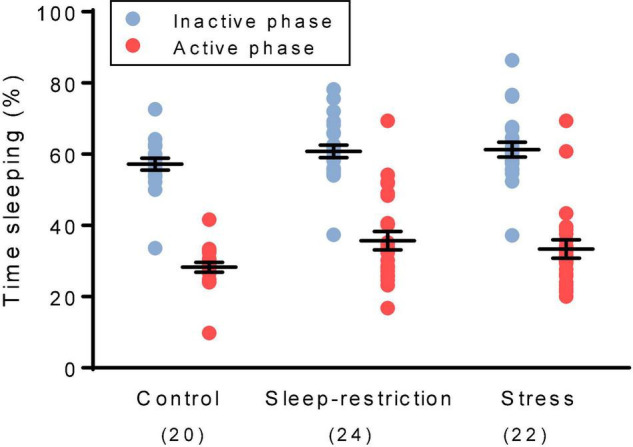
We assessed sleep duration during the recovery period beginning at P74. The main effect of phase was statistically significant (*p* < 0.001). Regardless of the condition, mice slept more in the inactive (light) phase than the active (dark) phase. The main effect of the condition and the condition × age interaction were not statistically significant. Each point represents the sleep duration in a single animal. Lines represent means ± SEMs for the number of mice indicated in parentheses.

We determined sleep duration toward the end of the recovery period by means of home-cage monitoring (Figure 3). As expected, the main effect of phase was statistically significant (*p* < 0.001) (Table 2), indicating that regardless of condition, mice slept more in the inactive (light) phase compared with the active (dark) phase. Neither the main effect of condition nor the condition × phase interaction was statistically significant (Table 2), indicating no long-term changes in sleep duration resulting from chronic stress or sleep restriction.

For comparison of the effects of stress and sleep restriction on sleep duration in WT and *Fmr1* KO mice, we included genotype as a variable (Supplementary Table 1). The genotype × phase interaction [*F*_(1_,_126)_ = 5.218, *p* = 0.024] was statistically significant. Regardless of the condition, *Fmr1* KO mice had decreased sleep in the inactive phase (light phase) compared with WT mice as previously reported (Sare et al., 2017a).

### Activity Response to a Novel Environment

**FIGURE 4:**
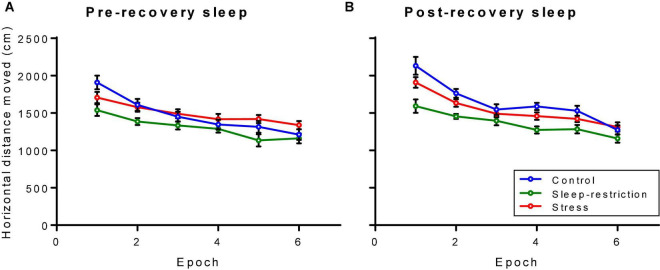
Distance traveled in an open field pre-recovery (P42) **(A)** and post-recovery (P84) **(B)**. The condition × epoch interaction was statistically significant (*p* < 0.001). Regardless of age, sleep-restricted mice were less active than control mice in epochs 1, 2, 4, and 5 (*p* < 0.001, *p* = 0.002, *p* = 0.025, and *p* = 0.030, respectively). Sleep-restricted mice were also less active than stress mice in epochs 1, 2, 4, 5, and 6 (*p* = 0.039, *p* = 0.016, *p* = 0.034, *p* = 0.011, and *p* = 0.05, respectively). Each point represents the mean ± SEM for 14, 23, and 22 mice in control, stress, and sleep-restricted groups, respectively.

We assessed activity by means of open-field testing (Figure 4). The main effect of age was statistically significant (*p* = 0.003) (Table 2), indicating that regardless of condition or epoch, mice at P84 (post-recovery) were more active than mice at P42 (pre-recovery). The condition × epoch interaction was statistically significant (*p* < 0.001) (Table 2). Regardless of age, sleep-restricted mice were less active than control mice in epochs 1, 2, 4, and 5 (*p* < 0.001, *p* = 0.002, *p* = 0.025, and *p* = 0.030, respectively). Sleep-restricted mice were also less active than the stress mice in epochs 1, 2, 4, 5, and 6 (*p* = 0.039, *p* = 0.016, *p* = 0.034, *p* = 0.011, and *p* = 0.05, respectively). These data indicate that in *Fmr1* KO mice, sleep restriction, but not stress, results in hypoactivity that persists even after recovery sleep.

Wild-type and *Fmr1* KO mice diverged with respect to activity in the open field (Supplementary Table 1). Pre-recovery (P42), the genotype × condition × epoch interaction was statistically significant [*F*_(10_,_645)_ = 2.413, *p* = 0.014], whereas post-recovery (P84), it was not [*F*_(10_,_645)_ = 1.239, *p* = 0.278]. In WT (pre-recovery), activity across epochs was similar for control and stress groups but was reduced in epochs 1–5 in sleep-restricted mice. In *Fmr1* KO mice, activity in the stress group was reduced in epoch 1 and increased in epochs 5 and 6 compared with control. In the sleep-restricted group, activity was reduced compared with control in epochs 1, 2, and 5. After recovery (P84), the genotype × epoch interaction was statistically significant [*F*_(5_,_645)_ = 2.836, *p* = 0.028] indicating that for WT mice, activity steadily decreased with time regardless of condition, whereas for *Fmr1* KO mice, activity decreased from epochs 1 to 3 but tended to rise in epochs 4 and 5. These results suggest that at P84, WT mice regardless of condition tend to adapt to the novel environment, whereas *Fmr1* KO mice do not.

### Anxiety-Like Behavior in Response to a Novel Environment

**FIGURE 5:**
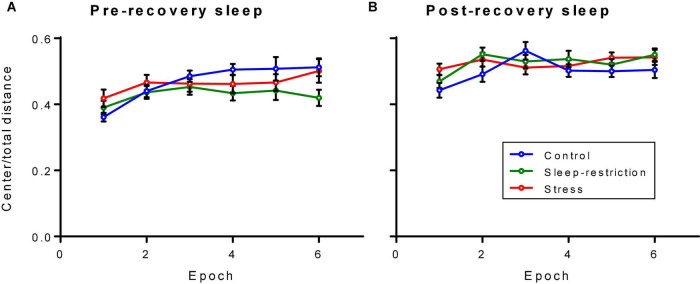
Ratio of distance traveled in the center to the total distance traveled in an open field arena pre-recovery (P42) **(A)** and post-recovery (P84) **(B)**. The condition × epoch interaction trended toward statistical significance (*p* = 0.059). Regardless of age, control mice traveled less relative distance in the center than stressed mice in epoch 1 (*p* = 0.051). Each point represents the mean ± SEM for 14, 23, and 22 in control, stress, and sleep-restricted groups, respectively.

We assessed anxiety-like behavior by measuring the ratio of distance traveled in the center of the open field to the total distance traveled (Figure 5). The main effect of age was statistically significant (*p* < 0.001) (Table 2). Regardless of the epoch or condition, older (P84, post-recovery sleep) mice traveled more relative distance in the center than mice at P42 (pre-recovery sleep) suggesting decreased anxiety-like behavior in older mice. The condition × epoch interaction trended toward statistical significance (*p* = 0.059) (Table 2). Regardless of age, control mice tended to travel less relative distance in the center compared with the stress group in epoch 1 (*p* = 0.051). This suggests that chronic stress resulted in decreased anxiety-like behavior in the initial response to a novel environment.

We compared these results in *Fmr1* KO mice with our reported results in WT mice by the inclusion of genotype as a variable (Supplementary Table 1). At both time points (i.e., pre- and post-recovery), the main effect of genotype was statistically significant [*F*_(1,129)_ = 12.72, *p* < 0.001 and *F*_(1,129)_ = 10.539, *p* = 0.001, respectively], indicating that *Fmr1* KO mice regardless of condition traveled more distance in the center of the field compared with WT. The shape of the time courses differed for condition in pre-recovery mice regardless of genotype [*F*_(10_,_645)_ = 2.008, *p* = 0.044], but this was a small effect. Overall, *Fmr1* KO mice regardless of condition or age demonstrated less anxiety-like behavior than WT.

### Repetitive Behaviors

**FIGURE 6:**
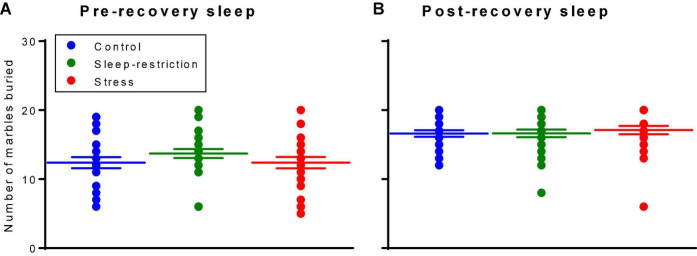
Marble burying assay assessed pre-recovery (P45) and post-recovery (P87). The main effect of age was statistically significant (*p* < 0.001). Regardless of the condition, mice buried more marbles at P87. Neither the condition × age interaction nor the main effect of condition was statistically significant. Each point represents the number of marbles buried by a single animal. Lines represent means ± SEMs for 23, 26, and 25 in control, stress, and sleep-restricted groups, respectively.

We assessed repetitive behaviors by means of a marble-burying assay (Figure 6). The main effect of age was statistically significant (*p* < 0.001) (Table 2). Regardless of the condition, older mice buried more marbles. Neither the main effect of condition nor the condition × age interaction was statistically significant. Our analysis of the effects of genotype on repetitive behavior indicates no statistically significant interactions or main effects either pre- or post-recovery (Supplementary Table 1).

### Social Behavior

**FIGURE 7:**
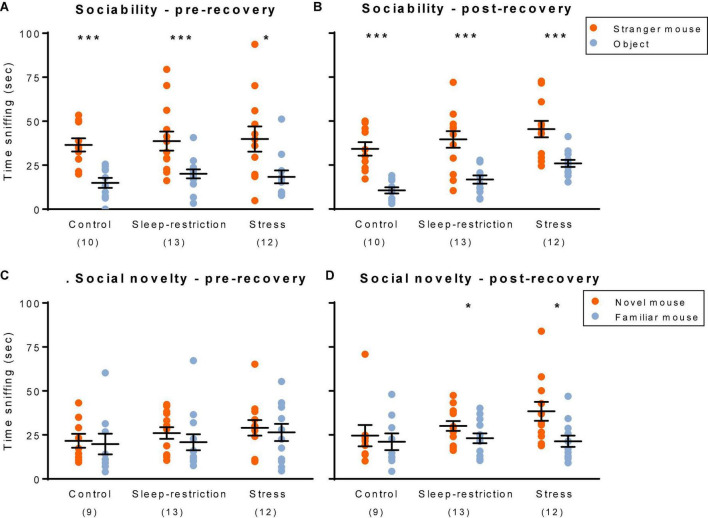
Social behavior: sociability **(A,B)** and social novelty **(C,D)**. Sociability pre-recovery (P48) **(A)** and post-recovery (P90) **(B)**. All three groups showed a statistically significant preference for the stranger mouse both pre- and post-recovery. Social novelty pre-recovery (P48) **(C)** and post-recovery (P90) **(D)**. Only the sleep-restricted and stress groups showed a preference for the novel mouse and only at the post-recovery time point. Sociability: each point represents the sniffing time for a single animal. Lines represent means ± SEMs for 10, 12, and 13 mice in control, stress, and sleep-restricted groups, respectively. Social novelty: each point represents the sniffing time for a single animal. Lines represent means ± SEMs for 9, 12, and 13 mice in control, stress, and sleep-restricted groups, respectively. Data were analyzed by means of paired *t*-tests comparing sniffing time for the stranger mouse vs. object pre- and post-recovery (sociability) and for novel vs. familiar mouse (social novelty). Significance levels are indicated on the figure as follows: *, 0.05 ≥ *p* ≥ 0.01; ***, 0.001 ≥ *p*.

We assessed social behavior by means of a three-chambered apparatus (Figure 7). First, to determine sociability, we compared how much time the test mouse interacted with either a stranger mouse or an object. In control *Fmr1* KO mice, preference for the stranger mouse over the object (Figures 7A,B) was statistically significant at both time points (i.e., pre- and post-recovery sleep). In sleep-restricted mice and stress mice, results were similar. We also assessed preference for social novelty by comparing how much time the test mouse interacted with either a novel mouse or a familiar mouse (Figures 7C,D). Control *Fmr1* KO mice did not demonstrate a statistically significant preference for social novelty at either time point. Sleep-restricted mice, however, did show a significant preference for social novelty post-recovery (*p* = 0.030), indicating some positive effect of the sleep restriction on social behavior. Stressed mice also showed a preference for social novelty post-recovery (*p* = 0.036). As with sleep restriction, the developmental stress protocol reversed the lack of preference for social novelty in *Fmr1* KO mice. In WT mice (Sare et al., 2019), both stress and sleep-restricted mice lost a preference for the novel mouse. Sociability was not affected in either genotype.

### Molecular Changes

**FIGURE 8:**
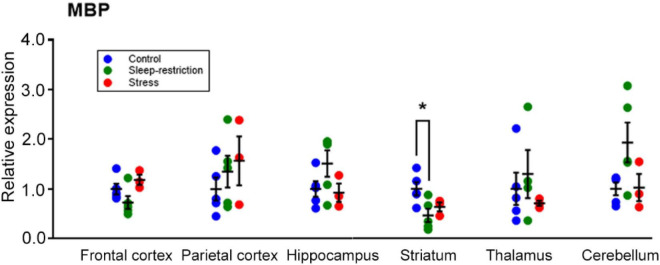
Regional expression of MBP and effects of sleep restriction and stress in the frontal cortex, parietal cortex, hippocampus, striatum, thalamus, and cerebellum. Data were normalized to controls for each region. The region × condition interaction was statistically significant (*p* = 0.008). Bonferroni-corrected *post hoc* tests showed that sleep restriction resulted in significantly decreased MBP in the striatum relative to controls (*p* = 0.027). Each point is the normalized value in a single animal. Lines represent means ± SEMs for 5 control, 4 stress, and 5 sleep-restricted mice. *p ≤ 0.05.

To correlate behavioral changes with molecular changes, we harvested mouse brains following the behavior testing period at P94 to perform Western blotting for candidate proteins in the frontal cortex. We examined pathways important for brain plasticity that have been implicated in response to sleep deprivation (Picchioni et al., 2014). Specifically, we examined pathways involved in cell death (LC3), cellular stress (JNK), circadian rhythm (CLOCK), activated microglia (Iba1), myelination (MBP), and transcription and translation (AKT, AMPK, CREB, ERK, GSK3, and mTOR) (Supplementary Figure 1). Whereas we did not detect any statistically significant main effect of condition for any protein assayed (Table 3), we found a trend for MBP (*p* = 0.078). We further probed MBP (Figure 8) and two other proteins (i.e., pAMPK and pS6) (Supplementary Figure 2) in all dissected regions (i.e., cerebellum, striatum, thalamus, hippocampus, frontal cortex, and parietal cortex) and analyzed results by means of ANOVA with region as a within-subject variable and condition as a between-subject variable (Table 4 and Supplementary Figure 2). For MBP, the region × condition interaction was statistically significant (*p* = 0.008). Bonferroni-corrected *post hoc* tests indicate that MBP in the sleep-restricted group was decreased in the striatum compared with controls (*p* = 0.027) (Figure 8). In the frontal cortex and cerebellum, we found trends for increased and decreased MBP, respectively, but these effects did not reach statistical significance. For p-AMPK, neither the condition × region interaction nor main effect of condition was statistically significant (Table 3 and Supplementary Figure 2). For pS6, we included the phosphorylation site as a within-subject variable. The phosphorylation site × region × condition interaction showed a trend toward statistical significance (*p* = 0.095) as did the main effect of condition (*p* = 0.080) (Table 4). Levels of pS6 (regardless of phosphorylation site) tended to be higher than control in both stress and sleep restriction groups (Supplementary Figure 2).

**TABLE 3:** ANOVA results Western blots: frontal cortex.

Protein	Interaction	Main effect	*F*_(df,error)_	*p*-Value	Partial η^2^
p4EBP1		Condition	*F*_(2_,_11)_ = 0.081	0.923	0.014
pAKT		Condition	*F*_(2_,_11)_ = 0.158	0.856	0.028
pAMPK		Condition	*F*_(2_,_11)_ = 2.671	0.113	0.327^‡^
CLOCK		Condition	*F*_(2_,_11)_ = 0.029	0.972	0.005
pCREB		Condition	*F*_(2_,_11)_ = 0.457	0.645	0.077^†^
pERK	Condition × band		*F*_(2_,_11)_ = 1.364	0.296	0.199^‡^
		Condition	*F*_(2_,_11)_ = 0.487	0.627	0.081^†^
		Band	*F*_(1_,_11)_ = 62.48	<0.001*	0.850^‡^
pGSK3	Condition × band		*F*_(2_,_11)_ = 0.503	0.618	0.084^†^
		Condition	*F*_(2_,_11)_ = 0.063	0.939	0.011
		Band	*F*_(1_,_11)_ = 83.90	<0.001*	0.884^‡^
Iba1		Condition	*F*_(2_,_11)_ = 0.879	0.442	0.138^‡^
pJNK	Condition × band		*F*_(2_,_11)_ = 0.692	0.521	0.112^†^
		Condition	*F*_(2_,_11)_ = 0.595	0.568	0.098^†^
		Band	*F*_(1_,_11)_ = 70.21	<0.001*	0.865^‡^
LC3		Condition	*F*_(2_,_11)_ = 0.058	0.944	0.010
MBP		Condition	*F*_(2_,_11)_ = 3.243	0.078^∼^	0.371^‡^
pmTOR Ser2448		Condition	*F*_(2_,_11)_ = 0.429	0.662	0.072^†^
pmTOR Thr2446		Condition	*F*_(2_,_11)_ = 0.833	0.461	0.131^†^
p-p90 RS6K		Condition	*F*_(2_,_11)_ = 0.037	0.964	0.007
p-p70 S6K		Condition	*F*_(2_,_11)_ = 0.298	0.748	0.051
pS6 235/236		Condition	*F*_(2_,_11)_ = 2.502	0.127	0.313^‡^
pS6 240/244		Condition	*F*_(2_,_11)_ = 2.188	0.159	0.285^‡^

*p ≤ 0.05.

*The symbol “∼” 0.10 ≥ p ≥ 0.05.*

*^†^Medium effect size based on partial η^2^.*

*^‡^Large effect size based on partial η^2^.*

**TABLE 4:** ANOVA results Western blots: multiple brain regions.

Protein	Interaction	Main effect	*F*_(df,error)_	*p*-Value	Partial η2
pAMPK	Region × condition		*F*_(7_,_41)_ = 19.203	0.792	0.093^†^
		Condition	*F*_(2_,_11)_ = 0.376	0.695	0.064^†^
		Region	*F*_(4_,_41)_ = 19.203	<0.001*	0.636^‡^
MBP	Region × condition		*F*_(9_,_48)_ = 2.914	0.008*	0.324^‡^
		Condition	*F*_(2_,_11)_ = 0.592	0.570	0.093^†^
		Region	*F*_(4_,_48)_ = 15.990	<0.001	0.254^‡^
pS6	Site × region × condition		*F*_(9_,_48)_ = 1.799	0.095^∼^	0.247^‡^
	Site × region		*F*_(4_,_48)_ = 3.503	0.012*	0.242^‡^
	Region × condition		*F*_(10_,_55)_ = 1.549	0.147	0.220^‡^
	Site × condition		*F*_(2_,_11)_ = 1.540	0.257	0.219^‡^
		Condition	*F*_(2_,_11)_ = 3.212	0.080^∼^	0.369^‡^
		Region	*F*_(5_,_55)_ = 2.141	0.074^∼^	0.163^‡^
		Site	*F*_(1_,_11)_ = 3.318	0.096^∼^	0.232^‡^

*Site is a measure of which phosphorylation site of pS6 is being measured.*

*p ≤ 0.05.

*The symbol “∼” 0.10 ≥ p ≥ 0.05.*

*^†^Medium effect size based on partial η^2^.*

*^‡^Large effect size based on partial η^2^.*

## Discussion

In this study, we examined the immediate and long-term behavioral effects and molecular correlates of chronic sleep restriction in *Fmr1* KO mice. We had hypothesized that sleep restriction would exacerbate abnormal phenotypes in *Fmr1* KO mice, but our results indicate that both the hyperactivity and the lack of preference for social novelty phenotypes were improved in sleep-restricted mice. Repetitive activity and anxiety-like activity were not measurably affected. We included a stress group, mice that were periodically prodded but not sleep-restricted, to try to control for the effects of the stressfulness of the sleep restriction procedure.

Sleep restriction is a much more subtle interference than sleep deprivation. Whereas we were unable to measure sleep duration during the remaining 21 h following the daily sleep restriction, it is likely that recovery sleep occurred during this time. It has been shown that recovery sleep following 3 h of sleep restriction (the maximum that is achievable in young neonatal animals due to sleep pressure) varies depending on age (Frank et al., 1998). Despite the subtle nature of the sleep restriction intervention, our previous study of sleep restriction in WT mice demonstrated short-term and long-term effects on behavior and molecular changes in WT mice (Sare et al., 2015, 2019), and these effects differed from the effects in the stress group. In this study, effects of sleep restriction in *Fmr1* KO mice also differed from the effects in the stress group, indicating that effects of sleep restriction cannot be explained by the stress of sleep restriction.

Fragile × behavioral phenotypes were not exacerbated by developmental sleep restriction as we had expected. One of the phenotypes we expected to observe was a reduction in the preference for social novelty as we had observed in WT mice (Sare et al., 2019). In contrast, our data show that preference for social novelty was improved in sleep-restricted mice following post-recovery sleep, a reversal of the phenotype we typically observed in *Fmr1* KO mice (Liu and Smith, 2009; Sare et al., 2017b). The fact that sleep restriction did not result in worsened behaviors and may have resulted in improvements in some behaviors merits further consideration. To be clear, we are not suggesting sleep deprivation as a therapeutic for FXS, but we are interested in the molecular correlates of our experimental protocol. Understanding the unfolding of these phenotypes may offer future treatment possibilities.

One of the targets that we found was altered following sleep restriction was the level of MBP, an essential component of myelin. Our results indicate that following recovery sleep, MBP was decreased in the striatum. These results indicate long-lasting alterations in myelin. Altered brain connectivity has been linked to social behavior and similarly linked to ASDs (Belmonte et al., 2004; Zhan et al., 2014).

Although it did not reach statistical significance, the mean values of sleep duration in sleep-restricted mice increased by 5.5% (regardless of phase). This is interesting because *Fmr1* KO mice sleep less than control mice in the inactive phase with a mean difference of 6.39% (Sare et al., 2017a). It may be that chronic sleep restriction during development resulted in a long-term improvement in sleep that results in an improvement in behavior. Indeed, in a preliminary experiment on sleep homeostasis in *Fmr1* KO mice, adult (P70) male mice were sleep-deprived for 24 h by a rotating bar. Following this period, we assessed recovery sleep and found that *Fmr1* KO mice had a greater sleep rebound than WT mice (Supplementary Figure 3). In this experiment, a daily 3 h session of sleep restriction may have led to recovery sleep in the ensuing 21 h. In brief, our manipulation may have resulted in a consolidation of sleep rather than a loss of sleep. Future experiments should perform EEG in *Fmr1* KO mice during development and ascertain the homeostatic recovery from sleep loss.

In conclusion, we observed that sleep restriction resulted in reductions in activity in *Fmr1* KO mice and improved social interactions. These surprising results should be examined to determine the underlying mechanism by which behavioral improvements occurred. We revealed evidence that chronic sleep restriction during development increased sleep duration, altered MBP in a regionally specific manner, and improved some behavioral phenotypes. These data may yield important insights into future treatments for FXS.

## Data Availability Statement

The raw data supporting the conclusions of this article will be made available by the authors, without undue reservation.

## Ethics Statement

The animal study was reviewed and approved by the National Institute of Mental Health Animal Care and Use Committee.

## Author Contributions

RS and CBS designed the research and wrote the manuscript. RS, AS, ML, AL, IL, CS, CH, and AM conducted the research. RS, AL, and CBS analyzed the data. All authors contributed to the article and approved the submitted version.

## Conflict of Interest

The authors declare that the research was conducted in the absence of any commercial or financial relationships that could be construed as a potential conflict of interest.

## Publisher’s Note

All claims expressed in this article are solely those of the authors and do not necessarily represent those of their affiliated organizations, or those of the publisher, the editors and the reviewers. Any product that may be evaluated in this article, or claim that may be made by its manufacturer, is not guaranteed or endorsed by the publisher.
