# Supplementary material for: Behavioral and Molecular Consequences of Chronic Sleep Restriction During Development in Fragile X Mice
Source: Front Neurosci. 2022 Jun 27;16:834890. doi: 10.3389/fnins.2022.834890 (PMC9271960; doi:10.3389/fnins.2022.834890)
Supplement: Supplementary file 1 [file Data_Sheet_1.PDF]

SUPPLEMENTAL MATERIAL

Supplemental Table 1. Repeated Measures ANOVA Results: Genotype and Condition Effects

	INTERACTION	MAIN EFFECT	$F_{(df,\ error)}$ VALUE	P-VALUE	PARTIAL ETA^2
Corticosterone					
P9	genotype x condition		$F_{(2,29)}$= 2.695	0.084	0.157‡
		genotype	$F_{(1,29)}$= 0.699	0.410	0.024
		condition	$F_{(2,29)}$= 0.721	0.495	0.047
P42	genotype x condition		$F_{(2,51)}$= 0.440	0.646	0.017
		genotype	$F_{(1,51)}$= 7.081	0.010*	0.122†
		condition	$F_{(2,51)}$= 2.529	0.090	0.090†
SLEEP					
Total Sleep Time	genotype x condition x phase		$F_{(2,126)}$ = 0.041	0.960	0.001
	genotype x phase		$F_{(1,126)}$ = 5.218	0.024*	0.040
	condition x phase		$F_{(2,126)}$ = 3.706	0.027*	0.056
	genotype x condition		$F_{(2,126)}$ = 1.377	0.256	0.021
		genotype	$F_{(2,126)}$= 1.388	0.241	0.011
		condition	$F_{(2,126)}$ = 1.928	0.150	0.030
		phase	$F_{(1,126)}$= 1774.5	<0.001*	0.934‡
OPEN FIELD					
PRE-RECOVERY					
Total distance	genotype x condition x epoch		$F_{(10,645)}$ = 2.413	0.014	0.036
	condition x epoch		$F_{(10,645)}$ = 3.603	<0.001*	0.053
	genotype x epoch		$F_{(5,645)}$ = 0.458	0.767	0.004
	genotype x condition		$F_{(2,129)}$ = 0.019	0.981	0.000
		genotype	$F_{(1,129)}$ = 12.72	0.001*	0.090†
		condition	$F_{(2,129)}$ = 6.637	0.002*	0.093†
		epoch	$F_{(5,645)}$= 148.74	<0.001*	0.536‡
Center/total	genotype x condition x epoch		$F_{(10,645)}$ = 0.258	0.978	0.004
	condition x epoch		$F_{(10,645)}$ = 2.008	0.044*	0.030
	genotype x epoch		$F_{(5,645)}$ = 0.901	0.462	0.007
	genotype x condition		$F_{(2,129)}$ = 1.184	0.309	0.018
		genotype	$F_{(1,129)}$ = 12.72	0.001*	0.090†
		condition	$F_{(2,129)}$ = 0.414	0.662	0.006
		epoch	$F_{(5,645)}$= 9.264	<0.001*	0.067†
POST-RECOVERY					
Total distance	genotype x condition x epoch		$F_{(10,645)}$ = 1.239	0.278	0.019
	condition x epoch		$F_{(10,645)}$ = 1.549	0.146	0.023
	genotype x epoch		$F_{(5,645)}$ = 2.836	0.028*	0.022
	genotype x condition		$F_{(2,129)}$ = 0.040	0.961	0.001
		genotype	$F_{(1,129)}$ = 12.24	0.001*	0.087†
		condition	$F_{(2,129)}$ = 15.91	<0.001*	0.198‡
		epoch	$F_{(5,645)}$= 125.57	<0.001*	0.493‡
Center/total	genotype x condition x epoch		$F_{(10,645)}$ = 1.327	0.214	0.020
	condition x epoch		$F_{(10,645)}$ = 1.521	0.131	0.023
	genotype x epoch		$F_{(5,645)}$ = 0.815	0.535	0.006
	genotype x condition		$F_{(2,129)}$ = 2.641	0.075	0.039
		genotype	$F_{(1,129)}$ = 10.54	0.001*	0.075†

		condition	$F_{(2,129)} = 0.494$	0.611	0.008
		epoch	$F_{(5,645)} = 11.62$	<0.001*	0.083†
<u>Marble Burying</u>					
Pre-recovery	genotype x condition		$F_{(2,141)} = 0.577$	0.563	0.008
		genotype	$F_{(1,141)} = 0.066$	0.798	0.000
		condition	$F_{(2,141)} = 1.712$	0.184	0.024
Post-recovery	genotype x condition		$F_{(2,141)} = 0.925$	0.399	0.013
		genotype	$F_{(1,141)} = 2.111$	0.148	0.015
		condition	$F_{(2,141)} = 0.861$	0.425	0.012

*, $P \leq 0.05$.

†, medium effect size based on partial eta^2.

‡, large effect size based on partial eta^2.

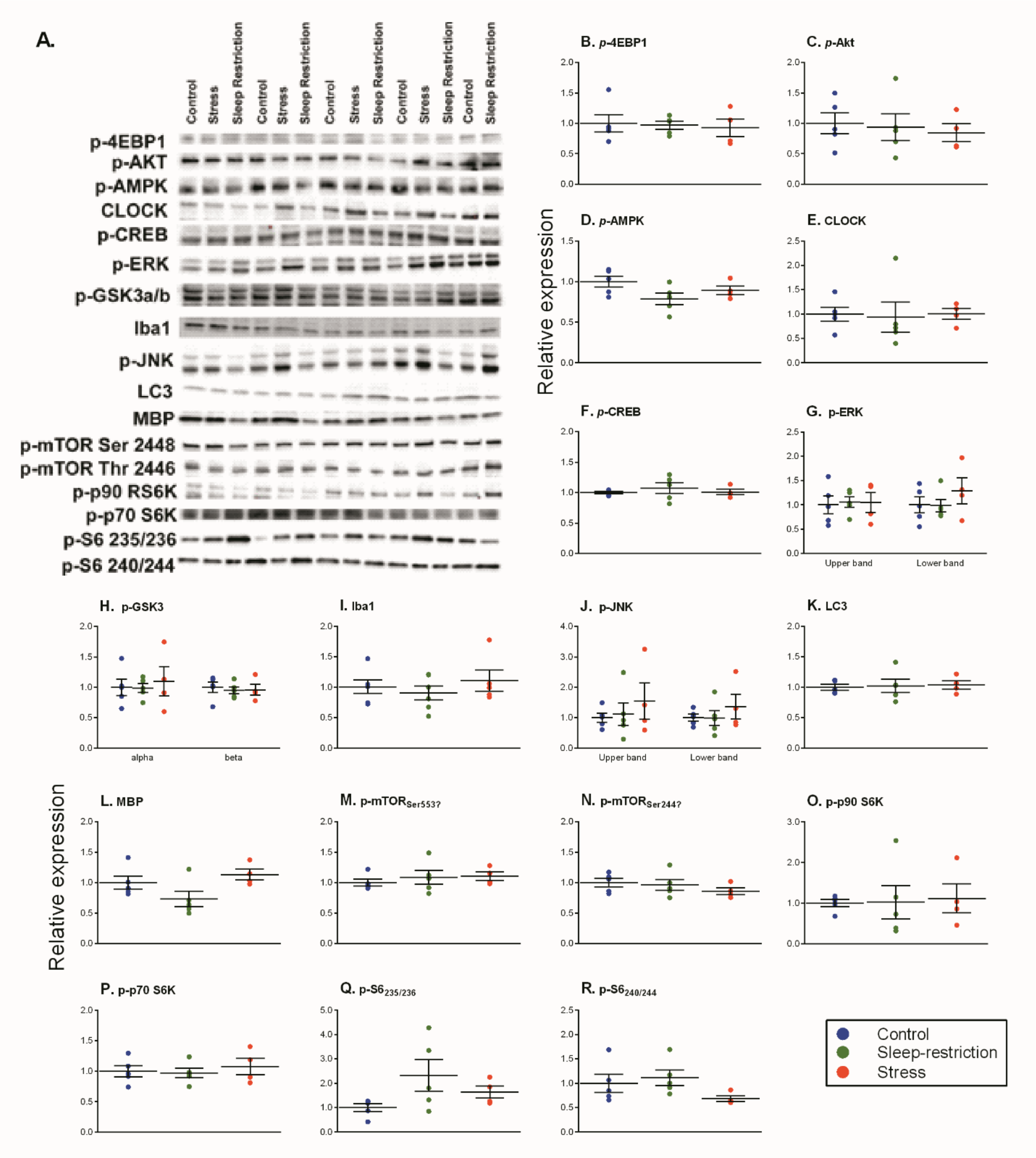

Supplemental Figure 1: Western blots. (A) Blots for all proteins (B-R). Normalized protein expression relative to controls. Each point is the normalized protein expression relative to the mean of controls for a single animal. Horizontal lines represent means ± SEMs for 5 control, 4 stress, and 5 sleep-restricted mice.

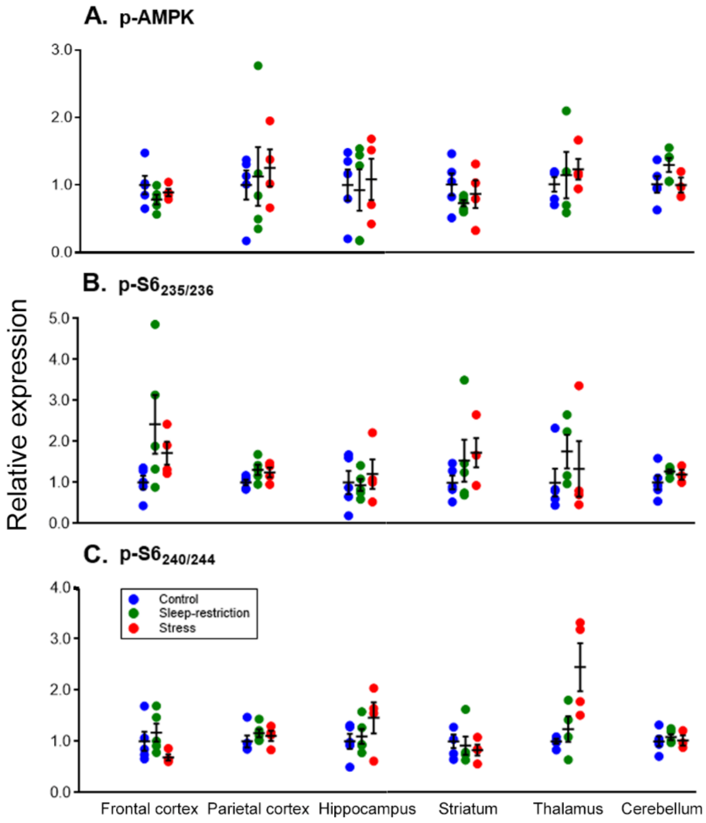

Supplemental Figure 2: Western blots. Regional expression of candidate proteins and effects of sleep-restriction and stress. Data were normalized to controls for each region. (A) For p-AMPK, only the main effect of region was statistically significant ($p<0.001$). There were no statistically significant main effects or interactions with condition. (B,C) For pS6, there was a trend toward a statistically significant site x region x condition interaction ($p=0.095$), though after Bonferroni correction, the only statistically significant *post-hoc* test was that stress increased pS6 240/244 in the thalamus relative to controls ($p=0.008$) and sleep-restricted ($p=0.024$) animals. There was also a trend toward a statistically significant main effect of condition ($p=0.080$) showing increased pS6 in both stress and sleep-restriction groups, though after Bonferroni correction, the *post-hoc* tests were not statistically significant. Each point is the normalized protein expression relative to the mean of controls in a single animal. Lines represent means ± SEMs for 5 control, 4 stress, and 5 sleep-restricted mice. Legend in C. applies to all panels.

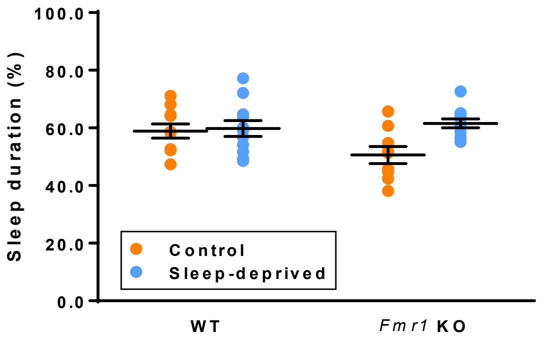

Supplemental Figure 3: We performed a separate experiment assessing recovery sleep following 24 hours of sleep-deprivation in adult (P70) WT and *Fmr1* KO male mice. Sleep-deprivation was achieved by means of a rotating bar (Pinnacle Technology, Lawrence, KS) in the bottom of the cage. Following the sleep-deprivation period, animals were transferred to new cages and sleep was assayed by the methods described previously. We did not analyze the first hour to allow for habituation to the new cage. We analyzed sleep duration during the subsequent five hours as percent time asleep. We found a statistically significant genotype x condition interaction ($F_{(1,37)}=8.786$; $p=0.047$). Bonferroni-corrected *post-hoc* comparisons show that only *Fmr1* KO mice demonstrated a statistically significant sleep rebound ($p=0.007$) indicating that sleep rebound is higher in *Fmr1* KO mice than WT mice. Each point represents the percent time asleep in a 5 h interval in a single animal. Lines represent means ± SEMs for 10 WT control mice, 11 WT sleep-deprivation mice, 9 *Fmr1* KO control mice, and 11 *Fmr1* KO sleep-deprivation mice.
